# Developing a shortened spine functional index (SFI-10) for patients with sub-acute/chronic spinal disorders: a cross-sectional study

**DOI:** 10.1186/s12891-024-07352-x

**Published:** 2024-03-26

**Authors:** Charles Philip Gabel, Antonio Cuesta-Vargas, Almir Vieira Dibai-Filho, Hamid Reza Mokhtarinia, Markus Melloh, Agnieszka Bejer

**Affiliations:** 1 Access Physiotherapy, PO Box 760, Coolum Beach, Queensland, 4573 Australia; 2 Department of Psychiatry and Physiotherapy, Faculty of Medicine, Malaga University, Malaga, Spain; 3 Federal University of Maranhao, Sao Luis, Maranhao Brazil; 4 Department of Ergonomics, University of Social Welfare and Rehabilitation Sciences, Tehran, Iran; 5 Queensland University of Technology, School of Public Health and Social Work, Brisbane, Australia; 6 Institute of Health Sciences, Medical College, Rzeszow University, Rzeszow, Poland; 7 The Holy Family Specialist Hospital, Rudna Mała 600, 36-060 Głogów Małopolski, Poland

**Keywords:** Spine, Musculoskeletal, Assessment, Patient-reported outcome measure, Functional limitation, Clinometric

## Abstract

**Background:**

Brief whole-spine patient-reported outcome measures (PROMs) provide regional solutions and future directions for quantifying functional status, evidence, and effective interventions. The whole-spine regional Spine Functional Index (SFI-25) is used internationally in clinical and scientific contexts to assess general sub-acute/chronic spine populations. However, to improve structural validity and practicality a shortened version is recommended. This study developed a shortened-SFI from the determined optimal number of item questions that: correlated with criteria PROMs being highly with whole-spine, moderately with regional-spine, condition-specific and patient-specific, and moderately-low with general-health and pain; retained one-dimensional structural validity and high internal consistency; and improved practicality to reduce administrative burden.

**Methods:**

A cross-sectional study (*n* = 505, age = 18-87 yrs., average = 40.3 ± 10.1 yrs) of sub-acute/chronic spine physiotherapy outpatients from an international sample of convenience. Three shortened versions of the original SFI-25 were developed using 1) qualitative ‘content-retention’ methodology, 2) quantitative ‘factorial’ methodology, and 3) quantitative ‘Rasch’ methodology, with a fourth ‘random’ version produced as a comparative control. The clinimetric properties were established for structural validity with exploratory (EFA) and confirmatory (CFA) factorial analysis, and Rasch analysis. Criterion validity used the: whole-spine SFI-25 and Functional Rating Index (FRI); regional-spine Neck Disability Index (NDI), Oswestry Disability Index (ODI), and Roland Morris Questionnaire (RMQ), condition-specific Whiplash Disability Questionnaire (WDQ); and patient-specific functional scale (PSFS); and determined floor/ceiling effect. A post-hoc pooled international sub-acute/chronic spine sample (*n* = 1433, age = 18-91 yrs., average = 42.0 ± 15.7 yrs) clarified the findings and employed the general-health EuroQuol-Index (EQ-5D), and 11-point Pain Numerical Rating Scale (P-NRS) criteria.

**Results:**

A 10-item SFI retained structural validity with optimal practicality requiring no computational aid. The SFI-10 concept-retention-version demonstrated preferred criterion validity with whole-spine criteria (SFI-25 = 0.967, FRI = 0.810) and exceeded cut-off minimums with regional-spine, condition-specific, and patient-specific measures. An unequivocal one-dimensional structure was determined. Internal consistency was satisfactory (*α* = 0.80) with no floor/ceiling effect. Post-hoc analysis of the international sample confirmed these findings.

**Conclusion:**

The SFI-10 qualitative concept-retention version was preferred to quantitative factorial and Rasch versions, demonstrated structural and criterion validity, and preferred correlation with criteria measures. Further longitudinal research is required for reliability, error, and responsiveness, plus an examination of the practical characteristics of readability and administrative burden.

## Background

Functional status measurement is frequently determined with patient-reported outcome measures (PROMs) as they provide optimal practicality, statistical coherence, and structural-validity [1]. For patients with spine disorders, there has been a progressive shift toward ‘whole-spine’ PROMs that measure status as a continuous functional kinetic-chain [2]. These have included static-PROMs, the Extended Aberdeen Spine Pain Scale (EASPS) [3], Functional Rating Index (FRI) [4], Spine Functional Index (SFI-25) [5], and the Computer Adaptive Testing (CAT) assessed Patient-Reported Outcomes Measurement Information System (PROMIS) for Physical Function (PROMIS-PF) [6, 7]. This whole-spine approach has high clinical relevance as a single, practical, psychometrically accurate, whole-spine PROM provides clinicians, researchers, and patients with a reduced administrative burden as multiple PROMs are no longer required for different regions and conditions [3, 8, 9]. This directly reduces the key barriers to PROM adoption [10, 11], complies with why a PROM is chosen and used under the essential nine pragmatic requirements [12, 13], and provides the capacity for a consistent spine single-score, broadened data-pooling, meta-analysis [14], and the capacity to demonstrate whether specific healthcare delivery is effective or not [15].

To balance the psychometrics, practicality, and cultural transferability, any whole-spine PROM must comply with the ‘Consensus-based Standards for the selection of Health status Measurement Instruments’ (COSMIN) standards [16]. The SFI-25 does this, being stringently developed and initially conference presented in 2004, with E-publication in 2013, with publication delays due to Journal submission processes and PhD by Portfolio requirements, with the official publication in 2019 affected by similar Journal-related delays [5]. This eventual peer validation permitted the inclusion of the SFI-25 within a whole-spine static-PROMs systematic review that considered the FRI and EASPS, where both had recognized concerns [8], but consequently did not include the PROMIS-PF. The FRI critiques were that it be used with caution till more robust high methodological quality studies are found to support its measurement properties [17], that it has item-construct deficiencies [18], and questionable ability to adequately represent whole-spine problems [8]. The EASPS, with 28–35 questions over four pages, is recognized as cumbersome with questionable COSMIN compliance [8].

The SFI-25 has had seven published validation studies [19–25], with a further comparative validation study under submission [26] and was most recently used in a chronic neck pain study [27]. These cultural-adaptation studies not only adapted and validated the SFI-25 for their specific linguistic and population requirements, but also performed criterion validity with multiple whole-spine, spine-region, general, and condition-specific populations. In each case, the SFI-25 was found preferable to the criteria PROMs that included the Neck Disability Index (NDI) [28], Oswestry Disability Index (ODI) [29], Roland Morris Questionnaire (RMQ) [30], and Whiplash Disability Questionnaire (WDQ) [31]. Additionally, suitable correlation was demonstrated with the patient-specific function scale (PSFS) [5] and EuroQuol-Index (EQ-5D) [19, 20], but less so with an 11-point pain numerical rating scale (P-NRS) [19] and the SF-36 PF scale [26]. However, the SFI-25’s structural validity was not unanimous with a shortened version recommended in most studies to improve practicality and structural validity.

The PROMIS-PF, using ‘CAT’ in varied spine-specific populations [32, 33], captures similar information to static-legacy PROMs [34, 35] but with greater efficacy and accuracy [6, 7, 36]. However, many populations lack the computing and internet accessibility necessary for PROMIS-PF, which, coupled with patient settings and computer literacy, must be considered [37]. Additionally, though content validity is sufficient, evidence quality in adult populations is low-moderate, particularly for single body areas and conditions, and elderly minority populations [38]. Further, minimal spine studies incorporated PROMIS-PF for its outcome measurement use, with substantial variability in domain validity between PROMIS-PF and criteria static-PROM [39]. Consequently, there remains a place and need for a simple-to-use, accurate, and practical whole-spine static-PROM with low administrative burden [12, 40].

The advocated methodologies to shorten PROMs are two-fold, qualitative and quantitative. Qualitative approaches use expert committee consensus with the ‘concept-retention’ method advocated for being judgmental and retaining the original PROMs theoretical domains [41]. Quantitative approaches use statistical methods, with ‘factorial’ and ‘Rasch’ the most common [1, 41]. This study aimed to: 1) develop a shortened-SFI for assessing spine functional status; 2) determine the correlation between the shortened-SFI and whole-spine criteria; 3) assess the correlation between the shortened-SFI and regional-spine, condition-specific, and patient-specific; criteria 4) investigate the correlation between the shortened-SFI and general-health and pain criteria; 5) ensure that the shortened-SFI retains the psychometric characteristics of one-dimensional structural validity, high internal consistency, and no floor/ceiling effect; and 6) enhance the practicality of the shortened-SFI to reduce administrative burden.

Accordingly, we hypothesized that: 1) the developed shortened-SFI will exhibit a high correlation with whole-spine criteria; 2) the correlation between the shortened-SFI and regional-spine, condition-specific, and patient-specific criteria will be moderate; 3) the correlation between the shortened-SFI and general-health and pain criteria will be moderate to low; 4) the psychometric properties of the shortened-SFI, including one-dimensional structural validity, high internal consistency, and absence of floor/ceiling effects, will be retained; and 5) practical enhancements made to the shortened-SFI will result in a reduction of administrative burden.

## Methods

### Study design

This cross-sectional study (*n* = 505) was conducted to shorten the SFI-25 to the SFI-10. All subjects provided written informed consent with the study approved by the Ethical Committee of the Universidade Federal do Maranhão (approval protocol number 4.284.203).

### Subjects

Participants were recruited from physiotherapy outpatients (*n* = 505, age = 18-87 yrs., av. = 40.3 ± 10.1 yrs., female = 50.5%, Table 1). There was no significant difference between the obtained SFI-10 scores by female (8.01 ± 6.14) and male (7.48 ± 5.60) (*p* = 0.317). *Inclusion criteria* were a medical/allied-health practitioner referral with a spine musculoskeletal disorder (MSD) diagnosis, sub-acute/chronic symptoms ≥ 2 weeks, age ≥ 18 years, written language competence, and informed written consent. *Exclusion criteria* were pregnancy, age < 18 years, and red-flag signs [19, 23].

**Table 1:** Demographics for all study participants

Study		*n*	Age x̄	Age *SD*	Female *n*	Female%	Cx	Tx	Lx	Multi
SFI-10 development and validation (Australian in English Language)		**505**	**40.1**	**13.0**	**255**	**50.5**	**149**	**48**	**350**	**53**
Bejer 2019 [19]	Polish SFI	225	45.7	16	135	60	92	38	114	19
Tonga 2015 [23]	Turkish SFI	285	45	1.0	207	73	129		151	5
Mokhtarinia 2018 [22]	Persian SFI	224	38.8	10.9	104	46.4	112	13	87	12
Freitas 2023 [26]	Brazilian SFI	194	29.1	8.5	136	70.1	38	60	96	
							36.3%	11.1%	55.7%	6.2%
	**TOTALS**	**1433**	**40.3 yrs Range 18-87 yrs**	**10.1 yrs**	**837**	**58.4%**	**520**	**159**	**798**	**89**

*SFI indicates* 25-item Spine Functional Index, *n* number, *x̄* mean, *SD* standard deviation, *%* percent, *Cx* cervical, *Tx* thoracic, *Lx* lumbar

* Subregion % values include multi-area individuals within each of their symptomatic regions making the total > 100%

The post-hoc international sample (*n* = 1433, age = 18-91 yrs., av. = 40.3 ± 10.1 yrs., female = 58.4%, Table 1) included retrospective de-identified data obtained with permission from the original researchers of three additional published SFI-25 cross-cultural adaptation studies [19, 22, 23] and a further data set from a completed MSc research study [26] that has progressed to journal submission.

## Measures

### The spine functional index (25 items)

*The SFI-25* has 25 item-questions with a 3-point response option ‘Yes’ (score = 1), ‘Partly/Sometimes’ (score = 1/2) and ‘No’ (score = 0). Item-questions have a biopsychosocial 60:40 item-question ratio [5, 42] with 15 ‘General’ (#1–15) and 10 ‘Region-specific’ (#16–25) item-questions. ‘Raw Score’ (0–25) totals from the summation of all item responses. The final score (0–100%: 0% = ‘worst possible’; 100% = ‘normal’/‘preinjury function’) is calculated by: [100-(Raw Score × 4)] [5], with two missing responses permitted and substituted with the average score of all responded item-questions [5].

### Functional rating index

*The FRI* has 10 item-questions with five short-descriptive response options (0–4 Likert visual NRS). ‘Raw Score’ (0–40) totals from the summation of all item responses. The final score, (0–100%: (0% = ‘no problem/pain’; 100% = ‘worst possible’) is calculated by: [Raw Score × 2.5] with one response permitted for substitution [4].

Each of the other spine-regional and general criteria PROMs are described in their original respective publications.

### Development and psychometric assessment of the SFI-10

*‘Development’* the *shortened* version of the SFI-25 was done through a-priori determination of the minimum number of item-questions necessary to retain structural validity and optimal practicality without a computational aid. The *minimum number* was guided by Spearman-Brown’s ‘k value’ [43, 44], the *optimal number* by completion/scoring-time, accuracy, and no computational aid being required [12, 45]. Additionally, one-dimensional structural integrity was required along with face, content and criterion validity (Pearson’s or Spearman’s r), plus internal consistency (Cronbach’s α:scale-level > 0.75; item-level > 0.65) [46, 47].

Four methodological approaches obtained the required optimal number of item-questions.

*Version A: qualitative ‘concept-retention’* [41] obtained consensus agreement using the “Ishikawa” qualitative process [48] from semi-structured interviews with ‘Expert’ (*n* = 7) and ‘Patient’ (*n* = 4) focus-groups [49]. The ‘Expert-group’ was four males and three females, included three physiotherapists, an occupational therapist, orthopedic specialist, registered nurse, and biostatistician. The ‘Patient-group,’ two males two female, paired for neck and back MSD.

*Version B: quantitative ‘factorial’* used exploratory factorial analysis (EFA) with polychoric correlation matrix and robust diagonally weighted least squares (RDWLS) extraction (Factor loading> 0.40) [50] to obtain the highest loading items. Retained factors were defined through parallel analysis with random exchange of observed data and robust promin rotation [51, 52]. Model adequacy used Kaiser-Meyer-Olkin (KMO > 0.70) and Bartlett’s sphericity tests (*p* > 0.05) from FACTOR software. The confirmatory factorial analysis (CFA) model used fit indices for: chi-square/degrees of freedom (chi-square/df < 3), root means square error of approximation (RMSEA<0.08; CI = 90%), comparative fit index (CFI > 0.90), and Tucker-Lewis index (TLI > 0.90) from R-Studio software with Lavaan and SemPlot packages [53].

*Version C: quantitative ‘Rasch’* extracted and confirmed the optimal-items through ‘Person Abilities’ and ‘Item Difficulties’ (preferred mean = 0.00); Personal separation reliability (PSR:cut-off> 0.70); one-dimensionality (Martin-Löf test:*p* > 0.05:*n* = 800 limit), and Principle Component Analysis (PCA) of Rasch-residuals Eigenvalues (cut-off = Linacare’s value< 2.0) [54]; ‘infit-outfit’ statistic elimination (range:0.5/0.7–1.3/1.5); item characteristic curves (ICCs); and thresholds proximity (three-response options crossover, with item difficulties ordering); ‘Wright-mapping’ (for item spacing and redundancy) [55]; ‘Algorithmic item-ranks’ and ‘Item-distances’; and Rasch corrected raw-scores (for person ability) [53].

*Version D: ‘Random’* selected 10 random computer-generated items.

*Validation’* selected the optimal shortened-version as that with the highest criterion-correlation (Pearson’s r) with whole-spine ‘Gold Standard’ criteria, the SFI-25 (*n* = 505, *r* > 0.95) and FRI (*n* = 343, *r* > 0.70) [47], supported by criterion validity cut-off scores (*r* > 0.50) with spine-regional instruments the NDI (*n* = 143), ODI (*n* = 194), RMQ (*n* = 31), and WDQ (*n* = 70), and the patient specific PSFS (*n* = 174). Full-sample structural validity was verified with EFA, CFA, and Rasch analysis, along with internal consistency (scale cut-off level:*α* > 0.75, item level:*α* > 0.65) and floor/ceiling effect from the percentage frequency for the highest/lowest scores (15% cut-off) [16].

A post-hoc pooled international sample (*n* = 1433) was analyzed to clarify structural validity, internal consistency, and floor/ceiling effect. Additionally, extracted Polish-study shortened-SFI scores (*n* = 225) [19] were compared with the SFI-25, spine-regional NDI (*n* = 49), ODI (*n* = 86), and general-health EQ-5D (*n* = 125) and pain P-NRS (*n* = 225); with the SFI-10 data referenced against the SFI-25. The Spearman r correlation coefficient (SCC) was used for non-normally distributed data.

The sociodemographic data and questionnaire scores used mean (x̄) and standard deviation (SD) in SPSS version 17 with significance:*p* < 0.05. The Kolmogorov-Smirnov test verified data-distribution. Factorial/Rasch analyses were blinded to minimize bias.

## Results

**Fig. 1:**
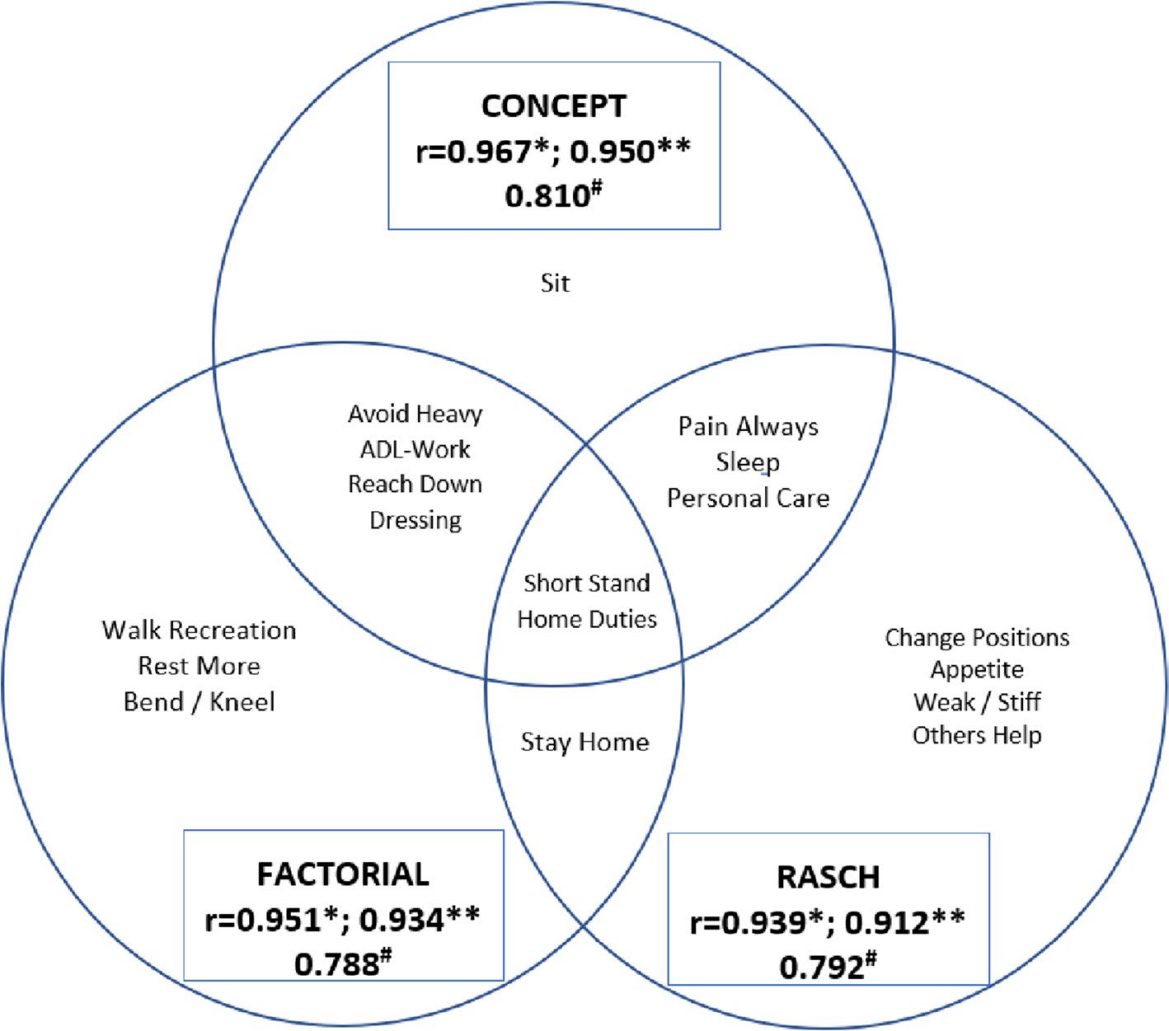
Reduction Approaches: Items and overlap of the three SFI-10 reduction methods and Pearson’s r correlation with: original SFI-25 (*n = 505, **n = 1433); and ^#^ = FRI (*n* = 343). Preferred SFI-10 was Concept version with the highest r value. Concept = qualitative concept-retention method; Factorial = factor analysis method; Rasch = Rasch analysis method. (Only two items were shared in all three methods. Concept shared the most items, then Factorial, then Rasch)

The ‘*Development’* indicated the *minimum number* of item-questions was *n* = 8 (Spearman-Brown k = 3.33). The *optimal number* of item-questions was *n* = 10, (from options of SFI-8, 10, 12 and 15 items), as this required no computational aid and retained the biopsychosocial 60:40 item-question ratio with six ‘General’ (#1–6) and four ‘Region-specific’ (#7–10) items (Fig. 1). The item-reduction and selection process confirmed face and content validity. The SFI-10 ‘Raw Score’ (0–10) is totaled from the summation of all item responses with the final score from: [100-(Raw Score × 10)], with one missing response and substitution permitted.

*‘Validation’* selected the 10-item qualitative concept-retention version as it: provided the highest Pearson’s r criterion-correlation with whole-spine criteria (SFI-25, *r* = 0.967, *n* = 505; FRI, *r* = 0.810, *n* = 343, Table 2); being supported by spine-regional and patient-specific criteria (*r* > 0.70, Table 3), except the NDI (*r* = 0.693) which approximated the *r* = 0.70 cut-off.

**Table 2:** Criterion validity comparing SFI-10 versions with the SFI-25 and FRI

		SFI-25	SFI-10			
Phase			Version A Qualitative ‘Concept’	Version B Quantitative ‘Factorial’	Version C Quantitative ‘Rasch’	Version D Computerized ‘Random’
**Australian sample (PCC)**	**SFI-25 (** ***n*** ** = 505)**		^#^ 0.967*	0.951*	0.939	0.955*
	**FRI (** ***n*** ** = 343)**	0.818	^#^ 0.810**	0.788**	0.792**	0.788**
**Pooled International sample (SCC) **[19, 22, 23, 26]	**SFI-25 (** ***n*** ** = 1433)**		^#^ 0.950*	0.934	0.912	0.938

*SFI* indicates Spine Functional Index, *PCC* Pearson’s Correlation Coefficient for normally distributed data, *FRI* Functional Rating Index

*PCC/SCC: *r* > 0.95 with the SFI-25; and **r > 0.70 with the FRI as the indicator of potential suitability to substitute for the SFI-25

^#^ PCC/SCC Highest value was the preferred version

**Table 3:** Criterion validity for the SFI-25 and SFI-10 from existing published research

PROM	Australian (PCC)		Polish [19] (SCC)		Turkish [23] (PCC)	Persian [22] (PCC)	Spanish [20] (PCC)	Chinese [24] (PCC)	Korean [21] (PCC)	Greek [25] (PCC)
	SFI-25	SFI-10	SFI-25	SFI-10	SFI-25	SFI-25	SFI-25	SFI-25	SFI-25	SFI-25
**SFI-25**		0.965 (*n* = 505)		0.943 (*n* = 225)						
		0.950 (*n* = 1433)								
**FRI**	0.832 (*n* = 343)	0.810 (*n* = 343)			0.52 (*n* = 285)			0.66 (*n* = 265)	0.57 (*n* = 60)	
**ODI**	0.818 (*n* = 194)	0.780 (*n* = 194)	0.800 (*n* = 86)	0.797 (*n* = 86)	0.71 (*n* = 151)			0.75 (*n* = 142)		0.58 (*n* = 60)
**RMDQ**	0.976 (*n* = 31)	0.893 (*n* = 31)				0.69 (*n* = 114)	0.79 (*n* = 133)		0.75 (*n* = 42)	0.70 (*n* = 60)
**NDI**	0.748 (*n* = 143)	0.693 (*n* = 183)	0.548 (*n* = 49)	0.321 (*n* = 49)	0.58 (*n* = 129)	0.57 (*n* = 124)		0.61 (*n* = 118)	0.53 (*n* = 26)	
**WDQ**	0.883 (*n* = 70)	0.837 (*n* = 70)								
**PSFS**	0.743 (*n* = 174)	0.734 (*n* = 174)								
**EQ-Index**			0.408 (*n* = 125)	0.532 (*n* = 125)			0.42 (*n* = 226)			
**P-NRS (SCC)**			−0.377 (*n* = 225)	−0.423 (*n* = 225)						

*SFI* indicates Spine Functional Index, *PCC* Pearson’s Correlation Coefficient for normally distributed data, *SCC* Spearman’s Correlation Coefficient for non-normally distributed data, *FRI* Functional Rating Index, *ODI* Oswestry Disability Index, *RMDQ* Roland Morris Disability Questionnaire, *NDI* Neck Disability Index, *WDQ* Whiplash Disability Questionnaire, *PSFS* Patient Specific Index, *EQ-Index* EuroQol Index, *P-NRS* Pain Numerical Rating Scale

The ten items selected were: ‘Avoid Heavy Jobs,’ ‘Pain/Problem,’ ‘Duties/Chores,’ ‘Sleep,’ ‘Personal Care,’ Daily Activity,’ ‘Dressing,’ ‘Sitting,’ ‘Standing,’ and ‘Reach/Bend Down’.

**Fig. 2:**
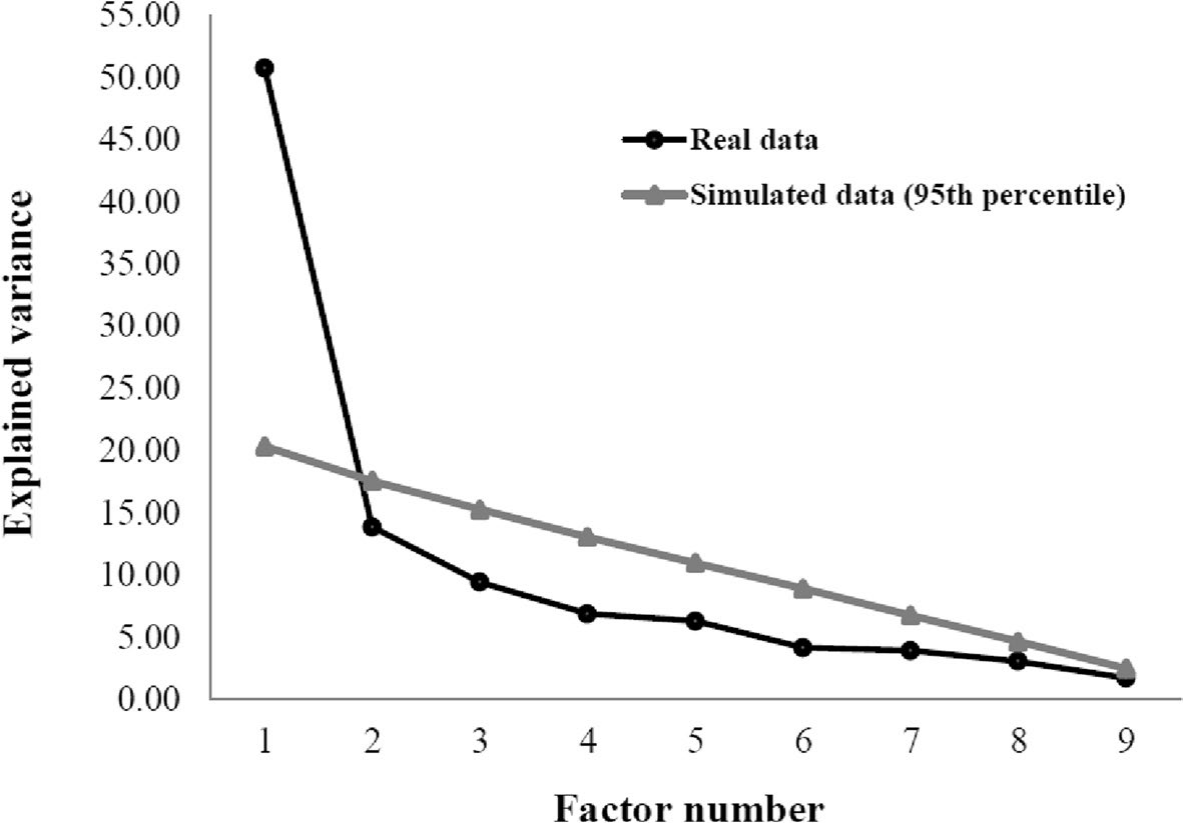
Representative Scree Plot for SFI-10 EFA (*n* = 505). Post-hoc retrospective pooled samples (*n* = 1433) are similar with inflection at point #2

**Fig. 3:**
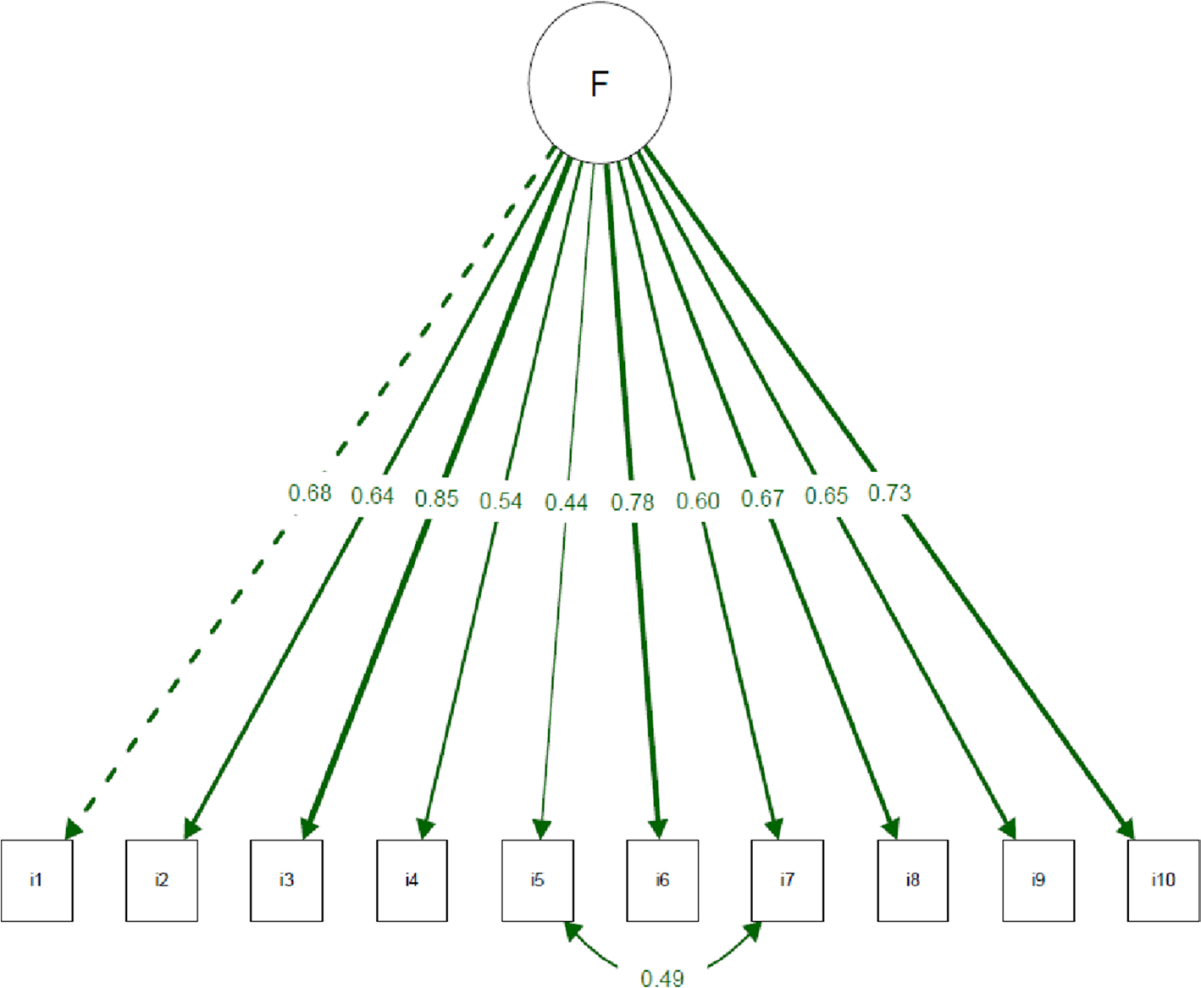
Representative Scree Plot for SFI-10 EFA (*n* = 505). Post-hoc retrospective pooled samples (*n* = 1433) are similar with inflection at point #2

Structural validity met the a-priori requirements. The EFA identified a one-dimensional structure (Fig. 2) (KMO = 0.79; Bartlett’s test *p* < 0.05). The CFA confirmed EFA with fit indices: chi-square/df = 2.06, CFI = 0.952, TLI = 0.939, RMSEA (90% CI) = 0.073 (0.049, 0.096) (Table 4). Appropriate factor loadings (> 0.40) were demonstrated between domains and items (Fig. 3).

**Table 4:** Structural validity determination from factorial (CFA) analysis

Study group	Chi-square/df	CFI	TLI	RMSEA (90% CI)
*n* = 505	2.06	0.952	0.939	0.073 (0.049, 0.096)
*n* = 1433	2.92	0.961	0.950	0.069 (0.062, 0.077)

*df* indicates degrees of freedom, *CFI* comparative fit index, *TLI* Tucker-Lewis index, *RMSEA* root means square error of approximation, *CI* confidence interval

*Rasch analysis* demonstrated adequate model fit (Table 5). ‘Person Abilities’ and ‘Item Difficulties’ indicated all tasks were within performance capacity, and PSR scores (0.71:0.79:0.75) exceeded the cut-off (> 0.70). One-dimensionality hypothesis (Martin-Löf test) was accepted (*p* > 0.50). Cut-off compliance was demonstrated for Rasch-residuals PCA (1.45–1.52:< 2.0), Infit-Outfit statistics (0.5–1.5), and item-difficulties (Table 5). Wright Map item-spacing and redundancy were acceptable, though some excess-spacing was present, but overall supported the selected item-shortening methodology. The ICC and Thresholds approximated a common point. Rasch corrected raw scores were completed (range:0–10). Rasch-analysis indicated the SFI-10 preserved the critical Rasch model-fit.

**Table 5:** Rasch analysis of the SFI-10 (*n* = 505 and *n* = 1433 are similar)

Item	SFI-25 Item #	Outfit		Infit		Item Difficulties	
		*n* = 505	*n* = 1433	*n* = 505	*n* = 1433	*n* = 505	*n* = 1433
1. Avoid Heavy Jobs	3.	1038	1167	0,967	1051	−0,675	− 0,535
2. Pain / Problem	6.	1001	1038	0,991	1022	−0,833	−0,570
3. Duties / Chores	10.	0,648	0,699	0,715	0,771	−0,071	0,093
4. Sleep	11.	1236	1277	1171	1174	−0,463	−0,416
5. Personal Care	12.	1231	0,992	1105	1005	1414	0,841
6. Daily Activity	13.	0,785	0,805	0,812	0,822	−0,229	−0,056
7. Dressing	16.	1040	0,971	0,993	0,963	0,606	0,247
8. Sitting	20.	0,969	0,996	0,952	0,948	−0,308	−0,239
9. Stand	22.	0,985	0,938	1005	0,951	0,015	−0,058
10. Reach/Bend Down	24.	0,848	0,890	0,877	0,918	−0,196	− 0,024

*SFI* indicates Spine Functional Index

Internal consistency exceeded the a priori cut-off (scale level *α* = 0.803, item level *α* > 0.65). No floor/ceiling effects were found as minimum/maximum scores were < 15%.

*Post-hoc analysis* of the pooled international sample (*n* = 1433) confirmed the ‘concept-retention’ findings with the highest Pearson’s r criterion validity compared with the whole-spine criteria (Table 2). The structural validity was one-dimensional where EFA used implementation of parallel analysis (KMO = 0.89, Bartlett’s test *p* < 0.05), and CFA fit indices approximated the main study (Table 4): chi-square/df = 2.92, CFI = 0.961, TLI = 0.950, RMSEA (90% CI = 0.069, 0.062, 0.077), with appropriate factor loadings (> 0.40) between domains and items. Rasch analysis approximated the main study and reinforced the one-dimensionality (Table 5). Internal consistency was high (scale level *α* = 0.863, item level *α* > 0.65) with no floor/ceiling effects.

The extracted Polish SFI-10 data criterion findings (Table 3) approximated the main study SFI-25 (*r* = 0.943 vs 0.965), ODI (*r* = 0.797 vs 0.780) except for the NDI (*r* = 0.321 vs 0.693). Similar correlations were found for the Polish SFI-25 with the spine-regional ODI, the EQ-5D and P-NRS criteria. The nine SFI-25 studies’ criteria findings were also comparable for the FRI, spine-regional, EQ-5D, and pain (Table 3).

## Discussion

The study’s essential aims were achieved with a shortened SFI-10 developed. Face and concept validity were demonstrated by the reduction process with the criterion and structural validity confirmed by the psychometric analysis. The SFI-10 correlated highly with whole-spine criteria PROMs, moderately with region-specific, patient-specific, and condition-specific, and moderate-low for general-health and pain. Practicality was improved by 60%, though completion/scoring time/errors require quantification. The SFI-10 qualitative ‘concept-retention’ version demonstrated higher criterion validity with whole-spine criteria than the quantitative ‘factorial’ and ‘Rasch’ versions, where both interestingly showed *lower PCC values* than the control/random (Table 2). Criterion validity was comparable with the FRI and slightly below the SFI-25 in the same sample and the original Australian SFI-25 study [5], but exceeded the Turkish [23], Korean [21] and Chinese [24] findings (Table 3).

Structural validity was unequivocally one-dimensional, being supported by factorial and Rasch analysis in the full *n* = 505 sample and the post-hoc international sample (*n* = 1433). This complied with previous research recommendations that factor structure be improved as, although a dominant single-factor was present, 6–8 factors were demonstrated [5, 20, 22, 23]. Spine-regional and patient-specific criteria correlations approximated the SFI-25 findings, but the RMQ and NDI were notably lower (Table 3). However, SFI-10 spine-regional and general-health criteria exceeded those of six SFI-25 studies [20–25] (Table 3).

Importantly, the SFI-10 retained the biopsychosocial 60:40 ratio conceptual model of general-versus-regional items [5, 42], which could not be maintained in the SFI-8, 12, and 15 item versions, each of which also required a computational aid. This biopsychosocial balance reduces risks of confounding ‘functional’ and ‘symptomatic’ change [56] while accommodating pain without potentially affecting responsiveness [57]. The increased SFI-10 practicality improved the scoring process without the need for a computational aid through a simple calculation of ‘× 10’ converting raw-scores to percentages [13, 45]. This should ensure lower administrative burden through reduced completion/scoring times [19, 40] and minimal potential errors [13], while complying with the essential nine pragmatic decisions for choosing and using a PROM [12]. In general, the popularity of short scales is explained by their need for reduced resources, particularly administrative burden and subsequent related costs [10, 40]. These findings reflect the two essential reasons for PROM shortening, practicality improvements and retaining validity and factor structure [16], as face, content, criterion, and structural validity must be retained [1, 46].

The preferred ‘concept-retention’ methodology supports similar PROM-shortening research where qualitative versions were superior to quantitative. This was demonstrated for the Quick-DASH (11-items) from the DASH (30-item) [41], though factor structure was not one-dimensional and practicality remained impaired as computational assistance was required. Similarly, concept-retention methodology produced the 10-item lower limb functional index (LLFI-10) from the LLFI-25 as a practical solution with one-dimensional validation in burns [58]. The 12-item Orebro Musculoskeletal *Screening* Questionnaire (OMSQ-12) improved the practicality of the original 21-item OM*Pain*SQ and retained the critical psychometric characteristics for biopsychosocial risk screening [59, 60]. This contrasts with a qualitative ‘author-determined’ OM*Pain*SQ-10 approach [61], where criterion validity was below the random version, as found in this study, and notably below the ‘concept-retention’ version [59]. The shortened NDI-5 combined qualitative and quantitative approaches, retained a one-dimensional structure [1, 56], and balanced psychometric and practical characteristics when compared to the 10-item version, the quantitative NDI-8 Rasch-version [57], and the NDI-7 factorial-version [1]. Various qualitative processes reduced the RMQ from 24 to 18 and 11 items [62], with the former, found preferable [62, 63]. However, no RMQ qualitative shortened version is available, and a computational aid remains necessary for all for practicality in calculating the scores of all RMQ versions. However, the question remains as to what is ‘the optimal minimum number’ of item-questions that provides a sufficiently broad representation of the required domains [64], and can this be represented by only five items as per the NDI-5 [1, 56].

This study demonstrated and reinforced that a qualitative approach does produce a shortened-PROM that has balanced the requirements for critical psychometric characteristics and one-dimensional structural validity while concurrently improving practicality. Very short scales, below 10-items, increase the measurement error from lower precision [64], hence the SFI-10 version appears an appropriate solution. Consequently, this concept-retention qualitative item-reduction process can be confidently applied to similar regional PROMs to facilitate their application in clinical and research settings.

### Study limitations and strengths

*Study limitations* include potential patient selection bias as recruitment was from primary contact and referred physiotherapy outpatients, consequently inpatient and community settings will need to be investigated. There is a lack of prospective data and repeated psychometric and practicality analysis. This leaves a knowledge gap in the test-retest reliability, responsiveness, and error scores, including both minimal detectable change and minimal clinically significant difference. Consequently, there is a need for longitudinal analysis, that includes patient-specific change, to clarify these psychometric properties. Further, the practical aspects of readability, missing responses, and administrative burden from completion and scoring times/errors must be quantified. Each of these latter limitations are now addressed in a subsequent study.

*Study strengths* included the large sample size and the clarification of findings in a further pooled international sample. Additionally, the SFI-10 development exceeded the minimal COSMIN standards and cut-off requirements. This incorporated the cross-sectional analysis and the pooled international sample from diverse populations with broad diagnoses.

## Conclusions

This study developed a shortened 10-item SFI-10 whole-spine PROM and verified structural validity through factorial and Rasch analysis, criterion validity and internal consistency with no floor/ceiling effects. The pooled MSD population of diverse age, culture, and clinical settings supported potential generalizability for outpatient settings, but inpatient and community settings require investigation. The improved practicality and unequivocal one-dimensional factor structure provided a summated score that is easily and rapidly determined without a computational aid. These attributes imply that the SFI-10 can be used in preference to the existing whole-spine and spine-regional PROMs in clinical and research settings. Further longitudinal research is currently underway to determine the critical psychometric characteristics of test-retest reliability, responsiveness, and error scores; and to quantify the practical characteristics of readability and administrative burden that include completion and scoring time/errors. Subsequently, a systematic review that includes the SFI-10 and published SFI-25 studies would further inform and clarify the clinimetric properties.

## Data Availability

The data that support the findings of this study are available on request from the corresponding author, HRM.

## Abbreviations

AP: Parallel analysis
AUC: Area under the curve
CFA: Confirmatory factor analysis
CFI: Comparative fit index
COSMIN: COnsensus-based Standards for the selection of health status measurement instruments
Df: Degrees of freedom
EASPS: Extended aberdeen spine pain scale
EFA: Exploratory factor analysis
EQ-5D: EuroQol 5-dimensions questionnaire
ES: Effect size
FRI: Functional rating index
G-NRS: Global-numerical rating scales (perceived function)
ICC: Intra-class correlation coefficient
KMO: Kaiser-meyer-olkin test
K-S: Kolmogorov-smirnov statistic
MCID: Minimal clinically important difference
MDC: Minimum detectable change
MSD: Musculoskeletal disorder
N: Number
NDI: Neck disability index
ODI: Oswestry disability index
PCA: Principal component analysis
PCC: Pearson’s correlation coefficient
P-NRS: Numerical rating scales (perceived pain)
PROM: Patient reported outcome measure
PSFS: Patient specific functional scale
PSR: Personal separation reliability
RDWLS: Robust diagonally weighted least squares
RMDQ: Roland morris disability questionnaire
RMSEA: Root means square error of approximation
ROC: Receiver operating curves
SCC: Spearman’s correlation coefficient
SD: Standard deviation
sec: Seconds
SEM: Standard error of the measurement
SFI-10: Spine functional index, 10-items
SFI-25: Spine functional index, 25-items
SPSS: Statistical package for the social sciences
SRM: Standard response mean
TLI: Tucker-lewis index
WDQ: Whiplash disability questionnaire
α: Cronbach’s alpha
