# Correction: Developing a shortened spine functional index (SFI‑10) for patients with sub‑acute/chronic spinal disorders: a cross‑sectional study

**DOI:** 10.1186/s12891-024-07622-8

**Published:** 2024-07-10

**Authors:** Charles Philip Gabel, Antonio Cuesta‑Vargas, Almir Vieira Dibai‑Filho, Hamid Reza Mokhtarinia, Markus Melloh, Agnieszka Bejer

**Affiliations:** 1 Access Physiotherapy, PO Box 760, Coolum Beach, Queensland 4573 Australia; 2 Department of Psychiatry and Physiotherapy, Faculty of Medicine, Malaga University, Malaga, Spain; 3 Federal University of Maranhao, Sao Luis, Maranhao, Brazil; 4 Department of Ergonomics, University of Social Welfare and Rehabilitation Sciences, Tehran, Iran; 5 Queensland University of Technology, School of Public Health and Social Work, Brisbane, Australia; 6 Institute of Health Sciences, Medical College, Rzeszow University, Rzeszow, Poland; 7 The Holy Family Specialist Hospital, Rudna Mała 600, 36‑060, Głogów Małopolski, Poland

**Correction: BMC Musculoskelet Disord 25, 236 (2024)**

10.1186/s12891-024-07352-x

Following publication of the original article [1], the authors would like to mark Dr. Charles Philip Gabel as deceased. Dr. Charles Philip Gabel passed away in January 2024. He was the main author in designing the study, writing the main file, and completing the article.

The original article [1] has been updated.
